# Comparison of a Concentrated Soy-Lecithin-Based Formulation and a Conventional Egg-Yolk-Based Formulation for Liquid Storage of Bull Semen at 0 °C: Effects on Sperm Quality, Antioxidant Capacity, and Fertility

**DOI:** 10.3390/biology15181577

**Published:** 2026-09-08

**Authors:** Peng Niu, Xiwen Deng, Xiaopeng Li, Fei Huang, Hong Chen, Xueyan Wang, Kai Hu, Di Fang, Qinghua Gao

**Affiliations:** 1 Key Laboratory of Tarim Animal Husbandry Science and Technology, Xinjiang Production & Construction Corps, Alar 843300, China; npn0805@126.com (P.N.); miexiaochi@163.com (X.L.); huangf21@126.com (F.H.); chenhong090588@163.com (H.C.); difang@taru.edu.cn (D.F.); 2 College of Animal Science and Technology, Tarim University, Alar 843300, China; 15569368760@163.com (X.D.); wangxueyan1016@163.com (X.W.); hk20012022@163.com (K.H.); 3 Key Laboratory of Livestock and Forage Resources Utilization in the Tarim Ba-sin Region, Ministry of Agriculture and Rural Affairs, Tarim University, Alar 843300, China

**Keywords:** treatment formulation, bull, semen preservation, antioxidant, fertilization ability

## Abstract

Semen diluents are crucial for the effective utilization of male germplasm resources and for enhancing the efficiency of artificial insemination (AI). However, reports on the in vitro low-temperature preservation of semen are still limited. This study comprised three separate experiments. Experiment 1 (semen storage): An ex vivo experiment using one ejaculate from each of five bulls, split into two aliquots per ejaculate, stored at 0 °C for 15 days, with repeated assessments of sperm quality (n = 5 biological replicates). Experiment 2 (IVF): An in vitro fertilization study using slaughterhouse-derived oocytes fertilized with day-15 stored semen from Experiment 1, conducted in five independent sessions (n = 5 sessions). Experiment 3 (AI): A non-randomized interventional field study involving 400 synchronized cows at China Hualing Ranch, allocated upon detection of estrus to receive either treatment or control semen (n = 400 cows). The primary outcome for Experiment 1 was total motility at day 15; for Experiment 2, blastocyst formation rate per oocyte; for Experiment 3, pregnancy rate at day 50 post-AI among all allocated cows. These findings suggest that the treatment formulation may offer advantages for liquid storage of bull semen at 0 °C.

## Simple Summary

This study compared two liquid-storage formulations for bull semen at 0 °C: a conventional egg-yolk-based diluent (control formulation) and a concentrated soy-lecithin-based diluent (treatment formulation). The treatment formulation showed more favorable values for some semen-quality outcomes during 15 days of storage. IVF and AI fertility findings are preliminary and require confirmation in larger, randomized trials.

## 1. Introduction

Artificial insemination (AI) technology maximizes the breeding potential of elite bulls and enables timely mating by overcoming temporal and geographical constraints. Semen diluents serve as a critical component in augmenting semen volume, facilitating cryopreservation, and enhancing reproductive efficiency [1]. The preservation of bull semen primarily relies on cryopreservation, while low-temperature storage serves as a supplementary option due to the inconsistent efficacy of existing diluent formulations in maintaining post-thaw semen viability [2,3]. Furthermore, current bull semen preservation protocols continue to encounter persistent challenges related to sperm viability, long-distance transportation, and cost-effectiveness.

The fundamental principle of cryopreservation technology centers on suppressing sperm metabolic activity through temperature reduction to extend viability duration [4]. However, the cryogenic environment induces detrimental alterations to spermatozoal physiological architecture and functionality, manifesting as diminished plasma membrane fluidity, mitochondrial dysfunction, and dysregulation of antioxidant defense systems. These cryoinjury-associated modifications may collectively compromise sperm viability parameters, reduce post-thaw survival rates, and potentially jeopardize genomic integrity [5,6,7]. In this study, bull semen was preserved at 0 °C using a treatment formulation, which mitigated cold-storage-related damage to sperm.

Recent years have witnessed substantial progress in refining diluent solution compositions through multidisciplinary investigations. For example, the addition of antioxidants, energy metabolism substrates, and cell protectants to egg yolk diluents has been demonstrated to significantly enhance sperm survival under low-temperature conditions [8,9]. However, these improvements still have limitations in practical applications, especially concerning biosafety risks, thermal sensitivity, and the reproducibility of results [10]. Therefore, the development of novel diluent formulations with improved efficacy and stability represents a critical research frontier, offering substantial scientific merit and practical implications for advancing bull semen preservation technologies [11,12].

This study presents an optimization of the KUNLUN No. I^®^diluent through the incorporation of soy lecithin and reduction of water volume, resulting in a modified formulation. This study aimed to compare the effects of this modified formulation and the conventional formulation on bull semen preservation at 0 °C.

## 2. Materials and Methods

### 2.1. Experimental Procedure

Sperm quality was assessed on days 0 (start of storage at 0 °C), 5, 10, and 15. Day 15 was selected as the final time point before the experiment began, based on practical farm scheduling and previous pilot observations, not on a prespecified biological threshold. Semen stored for 15 days was used for IVF and AI fertility testing.

#### 2.1.1. Experiment 1: Semen Storage Study

Design: Split-ejaculate, paired design. Each of five bulls provided one ejaculate, which was divided into two equal aliquots.

Groups: Control formulation (standard KUNLUN No. I^®^) (Tarim University, Alar City, Xinjiang, China) and treatment formulation.

Experimental unit: Ejaculate (bull).

Biological replicate: Bull (n = 5).

Technical replicate: Aliquot (2 per ejaculate), microscopic field (≥3 per slide), sperm cells (≥200 per field).

Repeated measure: Storage day (0, 5, 10, 15).

Primary outcome: Total motility (TM) at day 15.

Secondary outcomes: PM, VSL, VCL, ALH, BCF, acrosome integrity, plasma membrane integrity, MMP, DNA integrity, ultrastructure, antioxidant levels, gene expression.

#### 2.1.2. Experiment 2: In Vitro Fertilization Study

Design: Independent-session design using semen from Experiment 1 (day 15).

Groups: Control semen batch vs. treatment semen batch.

Experimental unit: IVF session (day).

Biological replicate: Session (n = 5).

Clustering factor: Semen batch (linked to bull/ejaculate from Experiment 1).

Primary outcome: Blastocyst formation rate per cultured oocyte.

Secondary outcomes: Cleavage rate per cultured oocyte, blastocyst rate per cleaved embryo.

#### 2.1.3. Experiment 3: Artificial Insemination Field Study

Design: Non-randomized interventional study with allocation upon estrus detection.

Groups: Control formulation semen vs. treatment formulation semen.

Experimental unit: Individual cow.

Biological replicate: Farm batch/insemination batch (n = 5 batches).

Clustering factors: Bull (semen source), semen batch, farm batch, inseminator.

Sample size: 400 cows enrolled; 210 allocated to control, 190 to treatment.

Primary outcome: Pregnancy rate at day 50 among all allocated cows (intention-to-treat).

Secondary outcome: Pregnancy rate among estrus-positive/inseminated cows (conditional analysis).

Reporting guideline: STROBE-Vet (for observational/interventional field studies) and ARRIVE 2.0 (for animal procedures).

#### 2.1.4. Primary, Secondary, and Exploratory Outcomes

The primary, secondary, and exploratory outcomes for each experiment were defined before data collection and analysis (protocol date: 6 February 2023). Experiment 1 primary outcome: Total motility (TM) at day 15 of storage. Secondary outcomes: Progressive motility (PM), VSL, VCL, ALH, and BCF at days 0, 5, 10, and 15; acrosome integrity; plasma membrane integrity; MMP; and DNA integrity at all time points. Exploratory outcomes: SEM/TEM ultrastructural assessment; antioxidant enzyme activities; ATP; MDA; ROS; and apoptosis-related gene expression. Experiment 2 primary outcome: Blastocyst formation rate per cultured oocyte. Secondary outcome: Cleavage rate per cultured oocyte. Experiment 3 primary outcome: Pregnancy rate at day 50 among all allocated cows (intention-to-treat denominator). Secondary outcome: Pregnancy rate among inseminated cows.

#### 2.1.5. Sample Size

No formal a priori sample-size calculation was performed for any experiment. The study is therefore exploratory. For Experiment 1, the sample size of five bulls was based on practical availability and conventional practice in bovine semen preservation studies. For Experiment 2, five IVF sessions were conducted to match the number of bulls/semen batches. For Experiment 3, 400 cows were enrolled based on the farm’s routine breeding schedule and semen availability. The ARRIVE checklist statement that ‘all animal samples meet the biological replication number (n = 3)’ was incorrect and has been removed.

#### 2.1.6. Multiplicity Control

No formal multiplicity adjustment (e.g., Bonferroni, Benjamini–Hochberg) was applied across the numerous outcomes and time points tested. This is a limitation. *p*-values for each outcome are reported unadjusted, and findings should be interpreted as exploratory rather than confirmatory.

Initially, we prepared the treatment formulation and the control formulation necessary for the study, which included both the treatment formulation and the control formulation. Fresh bull semen was mixed with diluent at a 1:4 (*v*/*v*) ratio (semen:diluent). The final working volume per aliquot was 20 mL. The final sperm concentration after dilution was 1 × 10^9^ sperm/mL. The diluted semen was transferred to sterile 50 mL conical centrifuge tubes (Corning^®^, 430829, Corning, NY, USA) and stored horizontally in a temperature-monitored refrigerator (Haier, DW-25L262, Haier Biomedical, Qingdao, China) at 0 °C ± 0.5 °C. Temperature was recorded every 30 min using a calibrated digital data logger (Elitech RC-5, accuracy ± 0.1 °C, Jiangsu Jingchuang Electronics Co., Ltd., Xuzhou, China). The acceptable temperature range was −0.5 °C to +0.5 °C; any deviation > 1 °C for >1 h was recorded as a protocol deviation (none occurred). For all assays, 10 uL of semen was used unless otherwise stated. Semen used for IVF and AI was stored for exactly 15 days at 0 °C before use.

### 2.2. Diluent Preparation

The patented diluent developed in our laboratory, referred to as KUNLUN No. I^®^, was utilized as the control formulation. The formulation details are provided in Table 1. Based on the solubility of the components in ddH_2_O, the maximum concentration factor was determined to be five-fold. Consequently, this experiment optimized the formulation of the KUNLUN No. I^®^ diluent to create a treatment formulation, with the detailed formulation provided in Table 2. This optimization aims to reduce costs and facilitate its application in production. The comparison between the control formula and the treatment formula is detailed in Table 2B.

### 2.3. Semen Collection, Dilution and Preservation

Five healthy Holstein breeding bulls (average age: 3.0 ± 0.5 years; range: 2.5–3.5 years) were used. All bulls were housed at the Tarim University research facility, fed a standard diet, and maintained in good body condition (BCS 3.0–3.5). Bulls had documented normal fertility histories (pregnancy rates > 60% in previous AI seasons) and no history of reproductive disease or antibiotic treatment within 3 months prior to semen collection. Semen inclusion thresholds: Initial motility > 70%, normal morphology > 80%, concentration > 1 × 10^9^ sperm/mL. All five bulls met these criteria; no bulls were excluded.

Each semen sample was immediately assessed for concentration and motility, and subsequently divided into two equal aliquots by laboratory technician A. Allocation of the two aliquots to control or treatment formulation was determined by a coin toss performed by technician B, who was not involved in semen collection. The sequence was generated immediately before allocation and was not blocked or stratified. Technician A, who prepared the diluents, was unaware of the allocation until after dilution was complete.

### 2.4. Assessment of Sperm Kinetic Parameters

The kinetic parameters of sperm were assessed utilizing computer-assisted sperm analysis (CASA, Hamilton Thorne, Beverly, MA, USA; MediPro, LSM900, ZEN Blue edition, Carl Zeiss, Jena, Germany). A volume of 10 μL of semen was introduced into the Makler counting chamber (Sefi Medical Instruments Ltd., Haifa, Israel) and examined under eight random fields (100× magnification, count at least 200 sperm cells per field of view) with the CASA system on a microscope stage maintained at a constant temperature of 38.5 °C [13]. The metrics that were recorded include total motility (TM), progressive motility (PM), straight-line velocity (VSL), curvilinear velocity (VCL), lateral head displacement amplitude (ALH), and beat or cross frequency (BCF).

### 2.5. Assessment of Sperm Acrosome Integrity

Semen samples underwent three washes with PBS that had been preheated to 37 °C, using a centrifugation process at 3000× *g* for a duration of 5 min. A semen sample measuring 30 µL was spread onto slides and allowed to dry in the air at room temperature (25 ± 1 °C). Subsequently, it was fixed using 4% paraformaldehyde (Sigma-Aldrich, St. Louis, MO, USA) for a duration of 30 min. The semen smears were washed with PBS and then stained with fluorescein isothiocyanate–peanut agglutinin (FITC-PNA, 12 µg/mL). The smears were incubated in a humidified chamber at 37 °C, away from light, for 30 min. Afterward, they were washed with PBS three times and stained with Hoechst (10 µg/mL) for a duration of 10 min [14]. Finally, the smears were washed three times with PBS, then mounted in the dark and imaged using a laser scanning confocal microscope (LSM 900, Carl Zeiss, Germany). For each smear, examine at least three fields and count a minimum of 200 sperm cells per field. The original images of the experiment can be found in Supplementary File S1—Acrosome Integrity.

### 2.6. Assessment of Sperm Plasma Membrane Integrity

The semen smears were washed with PBS and then stained with propidium iodide (PI, 5 µg/mL). The samples were incubated for 30 min in a dark, humid chamber at 37 °C, followed by three washes with PBS and staining with Hoechst (10 µg/mL) for 10 min [15]. Finally, the smears were washed three times with PBS, then mounted in the dark and imaged using a laser scanning confocal microscope. For each smear, examine at least three fields and count a minimum of 200 sperm cells per field. The original experimental images can be found in Supplementary File S1—Plasmalemma.

### 2.7. Assessment of Sperm Mitochondrial Membrane Potential (MMP)

The PBS was used to wash the semen smears, which were subsequently stained with JC-1 at a concentration of 4 µM [16]. JC-1 staining was performed using the JC-1 Mitochondrial Membrane Potential Assay Kit (Beyotime, C2006, Shanghai, China) according to the manufacturer’s protocol. Following this, the smears were positioned in a dark, humidified chamber maintained at 37 °C for a duration of 30 min. Subsequently, wash the smears three times with PBS, then mount them in a dark environment. Images should be captured using a laser scanning confocal microscope. For each smear, examine at least three fields and count a minimum of 200 sperm cells per field. The original images of the experiment can be found in Supplementary File S1—MMP.

### 2.8. Assessment of Sperm Chromatin Structure (Acridine Orange Method)

Note: The method described below measures the percentage of sperm showing red acridine orange (AO) fluorescence, which is indicative of denatured DNA. This is not the SCSA-validated DNA fragmentation index (DFI). We therefore report this outcome as ‘percentage of AO red-positive sperm’ rather than DFI.

Live sperm in suspension were diluted to 2 × 10^7^ sperm/mL in PBS. An acid denaturation step was performed by mixing 100 uL of sperm suspension with 200 uL of acid detergent solution (0.08 M HCl, 0.15 M NaCl, 0.1% Triton X-100, pH 1.2) for 30 s. Immediately thereafter, 600 µL of AO staining solution (6 µg/mL acridine orange in McIlvaine’s citrate-phosphate buffer, pH 6.0) was added [17].

Samples were analyzed within 5 min by flow cytometry (BD FACSCelesta) or confocal microscopy (Becton, Dickinson and Company, Franklin Lakes, NJ, USA). For microscopy, excitation was at 488 nm and emission was collected at 515–530 nm (green, double-stranded DNA) and >630 nm (red, single-stranded/denatured DNA). A minimum of 500 sperm were counted per sample. The percentage of sperm showing red fluorescence (denatured DNA) was calculated as (red sperm/total sperm counted) × 100.

Controls: Positive control—sperm treated with 1 mM H_2_O_2_ for 30 min at 37 °C (expected > 40% red). Negative control—fresh semen with known low DNA damage (expected < 5% red). Image analysis: Manual scoring using ImageJ (version 1.54). Biological replicates: 5 bulls × 2 aliquots × 4 days = 40 samples. Technical replicates: 3 slides per sample. Assessor masking: Coded slides, scorer blinded to group.

### 2.9. Scanning Electron Microscope (SEM) Analysis

Samples were preserved in a 2.5% glutaraldehyde solution (prepared in 0.1 M PBS, pH 7.4) at 4 °C for a duration of 48 h, subsequently subjected to three gradient rinses with PBS buffer (with each centrifugation occurring at 1000× *g* for 5 min). Subsequently, the samples were fixed again in a 1% osmium tetroxide solution (also dissolved in 0.1 M PBS, pH 7.4) at 4 °C for 2 h. Ethanol dehydration was subsequently carried out sequentially with concentrations of 30%, 50%, 70%, 90%, and 100%, with each stage lasting for 10 min. The samples that had been dehydrated were dried utilizing a CO_2_ critical point dryer (Leica EM CPD300, Leica Microsystems, Wetzlar, Germany) and later coated with a 5 nm layer of a gold–palladium alloy through the Gatan 682 PECS II system (Gatan, Inc., Pleasanton, CA, USA) [18]. Finally, the samples were examined using a Thermo Fisher Scientific Apreo S scanning electron microscope (Thermo Fisher Scientific Inc., Waltham, MA, USA) at the Central Laboratory of Tarim University. Representative images were captured from at least three distinct regions of each specimen under each experimental treatment for qualitative assessment, utilizing an accelerating voltage of 5 kV and achieving a resolution of 3 nm at the indicated magnifications (×5000 for overview; ×20,000 for detail). The instrument was upgraded from Hitachi Regulus 8230 to Thermo Fisher Apreo S in 2024; all images in this study were acquired using the Apreo S. The original images of the experiment can be found in Supplementary File S1—SEM.

### 2.10. Transmission Electron Microscopy (TEM) Analysis

The fixation treatment of semen samples adhered to the established sample preparation protocol for SEM, utilizing a dual fixation method involving glutaraldehyde and osmium tetroxide. Following fixation, the samples underwent gradient infiltration with epoxy resin Epon 812, incubated at 37 °C for 12 h, at 45 °C for an additional 12 h, and cured at 60 °C for 24 h. The hardened samples were sectioned using a Leica UC7 ultramicrotome (Leica, Wetzlar, Germany), with a slice thickness calibrated to 70 nm. The sectioned samples were initially placed in a concentrated solution of uranyl acetate, which was dissolved in ethanol, for 8 min in the absence of light. Subsequently, they were rinsed with 70% ethanol and ultrapure water. Thereafter, the samples were stained with a 2.6% lead citrate solution under carbon dioxide-free conditions for 8 min, rinsed again with ultrapure water, and dried at room temperature in a copper grid box [19]. Finally, the samples were examined using a Hitachi HT7800 transmission electron microscope (Hitachi, Tokyo, Japan) at the Central Laboratory of Tarim University. For each experimental treatment, representative images were captured from a minimum of three distinct regions of each specimen for qualitative assessment, employing an accelerating voltage of 80 kV and a CCD resolution of 0.38 nm. The instrument was upgraded from JEOL JEM-1400Flash to Hitachi HT7800 in 2024; all images in this study were acquired using the HT7800 at 80 kV. The original images of the experiment can be found in Supplementary File S1—TEM.

### 2.11. Measurement of Antioxidative Levels in Whole Semen-Extender Mixtures

Important note: Sperm were not separated from the extender before analysis. The measurements therefore reflect the whole semen-extender mixture, not intrinsic sperm ATP or antioxidant activity alone. Cell-free extender blanks were measured for both formulations and subtracted from the raw values.

Semen samples were gently mixed, and 100 uL of raw diluted semen was diluted with 900 uL of physiological saline (0.9% NaCl) at a 1:10 (*v*/*v*) ratio. The mixture was homogenized by vortexing for 10 s and equilibrated at 25 °C for 15 min. No centrifugation or washing was performed.

Cell-free extender blanks: Both control and treatment extenders (without sperm) were prepared identically and measured in parallel. Blank absorbance values were subtracted from sample values.

Seminal plasma controls: Fresh seminal plasma (before dilution) from each bull was measured as a reference.

Protein assay: Total protein concentration in each semen-extender mixture was measured using the BCA Protein Assay Kit (Pierce, 23225, Waltham, MA, USA). All antioxidant and ATP results are reported per mg of total protein (or per 10^6^ sperm, where sperm concentration was measured by hemocytometer).

Assay details: ATP (Beyotime, S0026, luciferase method, 560 nm, Shanghai, China), T-AOC (Nanjing Jiancheng, A015-1-2, ABTS method, 593 nm, Nanjing, China), MDA (Nanjing Jiancheng, A003-1-2, TBA method, 532 nm), ROS (Beyotime, S0033, DCFH-DA method, 530 nm excitation, 590 nm emission, Shanghai, China), *CAT* (Nanjing Jiancheng, A007-1-1, ammonium molybdate method, 520 nm), *SOD* (Nanjing Jiancheng, A001-1-2, WST-1 method, 450 nm), GSH-Px (Nanjing Jiancheng, A005-1-2, NADPH method, 340 nm). Each sample was measured in technical triplicate. Enzyme activity was calculated using the manufacturer’s standard curve and formula, corrected for dilution factor (10×) and blank subtraction.

### 2.12. Total RNA Extraction and cDNA Synthesis

Total RNA was isolated from spermatozoa utilizing the RNAprep Pure Animal Tissue Total RNA Kit (Tiangen, DP431, Beijing, China). Sperm purification: Before RNA extraction, sperm were purified from extender components by centrifugation at 3000× *g* for 10 min at 4 °C, followed by two washes in PBS. Somatic cell exclusion: The absence of somatic cells was verified by microscopy; any samples with visible epithelial or leukocyte contamination were excluded.

RNA quantification and quality: RNA concentration was measured using a NanoDrop One spectrophotometer (Thermo Fisher, Waltham, MA, USA). Total RNA input: 500 ng per reverse transcription reaction. RNA integrity: Assessed by agarose gel electrophoresis (1.5%); all samples showed intact 18S and 28S bands. RNA purity: A260/A280 ratios ranged from 1.8 to 2.1; A260/A230 ratios > 1.5.

cDNA synthesis was performed using the FastKing one-step genomic cDNA removal first-strand synthesis premix kit (Tiangen, KR118, Beijing, China). The reaction mixture comprised 4 uL of 5x FastKing-RT SuperMix, 2 uL of RNA template (500 ng), and 14 uL of RNase-free ddH_2_O. Reaction conditions: 42 °C for 15 min, 95 °C for 3 min.

#### qPCR Quality Control

No-template controls (NTC): Included for each gene; all NTCs showed no amplification (Ct > 40).

No-reverse-transcription controls (No-RT): Included for each sample; all No-RT controls showed Ct > 35, confirming minimal genomic DNA contamination.

Melt-curve analysis: Single peak confirmed for all primer pairs; no primer-dimer artifacts detected.

Primer specificity: Verified by agarose gel electrophoresis of PCR products (single band at expected size) and melt-curve analysis.

Primer efficiency: Determined by standard curve (5-fold serial dilutions of pooled cDNA); efficiencies ranged from 95% to 105% (R^2^ > 0.98).

Reference gene stability: *GAPDH* stability across storage times and groups was confirmed by geNorm algorithm (M = 0.32, V < 0.15); *GAPDH* was selected as the single reference gene.

Calibrator: Day 0 control aliquot from Bull_01.

Biological replicates: 5 bulls × 2 aliquots × 4 days = 40 samples.

Technical replicates: 3 per sample; technical replicates were averaged before statistical analysis.

### 2.13. Primer Design and Synthesis

Primers were created using Primer Premier 5.0 software based on the relevant gene sequences available in GenBank, with *GAPDH* serving as an internal reference gene, as shown in the primer sequences in Table 3.

### 2.14. PCR Detection

The SYBR Green PCR kit (QIAGEN, Hilden, Germany) was utilized to conduct real-time quantitative PCR, following the guidelines provided by the manufacturer. The reaction mixture was prepared with a total volume of 10 μL, which included 1 μL of cDNA, 1.5 μL each of the forward and reverse primers, 5 μL of 2 × SYBR Green Master Mix, and 1 μL of ddH_2_O. The reaction conditions started with an initial phase at 95 °C lasting 2 min, and this was succeeded by a cycle consisting of 95 °C for 5 s, followed by temperatures of 55/59 °C for 10 s. This sequence was repeated for a total of 40 cycles. For each sample and gene, three technical replicates were conducted, and the 2^−ΔΔCt^ method was employed to assess the relative expression levels of the target genes.

### 2.15. Fertilization and Embryonic Development

IVF tests were carried out in five independent replicates (sessions), each on a separate day. Ovaries from Holstein cows were collected from a local slaughterhouse (Alar City Abattoir, Xinjiang) and transported to the laboratory in sterile saline at 25–30 °C within 2 h. Cumulus–oocyte complexes (COCs) were aspirated from follicles (2–8 mm) using an 18-gauge needle. COCs with intact cumulus layers were selected and washed three times in TL-HEPES medium (Gibco, 12313-013, Waltham, MA, USA). Groups of 50 COCs were placed in four-well dishes (Nunc, 176740, Roskilde, Denmark) containing 500 uL of maturation medium per well (TCM-199 supplemented with 10% fetal bovine serum (Gibco, 10099141, Waltham, MA, USA), 0.01 IU/mL FSH, 0.01 IU/mL LH, 1 µg/mL estradiol, and 0.1 mM cysteamine; Sigma-Aldrich, M4530, St. Louis, MO, USA), overlaid with mineral oil (Sigma-Aldrich, M5904, St. Louis, MO, USA), and incubated at 38.5 °C, 5% CO2, 100% relative humidity for 22–24 h [20].

Following maturation, cumulus cells were partially removed by pipetting in fertilization medium (Tyrode’s albumin lactate pyruvate, TALP; Gibco, A1435102, Waltham, MA, USA). Matured oocytes were co-incubated with swim-up purified semen at a final concentration of 1 × 10^6^ sperm/mL in 500 uL fertilization drops under mineral oil. Fertilization was carried out at 38.5 °C, 5% CO_2_, 100% humidity for 18 h.

After fertilization, putative zygotes were washed three times in embryo development medium (SOFaa; Sigma-Aldrich, S2920, St. Louis, MO, USA) and cultured in 500 uL drops (groups of 25–30 embryos per drop) under mineral oil at 38.5 °C, 5% CO2, 5% O2, 90% N2, 100% humidity. The medium was changed every 48 h. Cleavage was assessed at 24 h post-insemination (hpi). Blastocyst development was assessed at day 6 (144 hpi).

### 2.16. Assessment of Pregnancy Rate After AI

A total of 400 healthy Holstein cows from China Hualing Ranch were assessed for eligibility. Cows were synchronized using the GnRH-PG-GnRH protocol (see Section Estrus Synchronization Details for details) [21]. Allocation was not randomized. Upon detection of estrus, the herd manager assigned cows to receive either control or treatment semen based on the order of estrus detection and semen batch availability: cows detected in estrus on odd-numbered days within each batch received control semen; cows detected on even-numbered days received treatment semen. This allocation rule was predetermined but not randomized. Important potential confounders include parity, lactation status, body condition score, days postpartum, and inseminator. These variables were recorded and included in the statistical model.

Each cow received a single insemination dose of 0.25 mL straws containing 2 × 10^7^ total sperm (final concentration: 8 × 10^7^ sperm/mL). AI was performed 8–12 h after detected estrus (which occurred 24–48 h after the second GnRH treatment) by three experienced inseminators (Inseminator A, B, and C). Semen was deposited into the uterine body using a disposable sheath catheter (Minitube, 12345-AI, Tiefenbach, Germany). The identity of the bull, semen batch, and inseminator were recorded for each cow.

#### Estrus Synchronization Details

Estrus synchronization was performed using the 0–7–9-day GnRH-PG-GnRH protocol. Day 0: GnRH (Receptal^®^, Merck Animal Health, gonadorelin diacetate tetrahydrate, 100 ug, intramuscular, Madison, NJ, USA). Day 7: Prostaglandin F2alpha (Estrumate^®^, MSD Animal Health, Rahway, NJ, USA, cloprostenol sodium, 500 ug, intramuscular). Day 9: Second GnRH (100 ug, intramuscular, Rahway, NJ, USA). Estrus detection was performed by trained farm staff at 06:00 and 18:00 daily beginning on Day 8 using visual observation and tail paint. Cows showing standing estrus were allocated to a semen group as described above and inseminated 8–12 h after detected estrus.

Study sequence: (1) 400 cows were enrolled and assessed for eligibility; (2) 400 cows received synchronization; (3) 400 cows were monitored for estrus; (4) upon estrus detection, cows were allocated to control (n = 210) or treatment (n = 190) based on the predetermined rule; (5) all allocated cows were inseminated; (6) pregnancy was diagnosed by rectal palpation at day 50 post-AI.

Inclusion criteria: (1) Holstein dairy cows aged 2–8 years; (2) body condition score 2.5–4.0; (3) clinically healthy with no signs of mastitis, metritis, or other reproductive disorders; (4) at least 50 days postpartum for lactating cows; (5) no hormone treatment within 60 days prior to enrollment. Exclusion criteria: (1) Pregnancy at enrollment (n = 8); (2) failure to respond to synchronization (no estrus signs by day 12, n = 12); (3) withdrawal by farm manager due to health issues (n = 0). All criteria were defined before the study began.

### 2.17. Statistical Analysis

All analyses were conducted using R version 4.3.1 (R Core Team, Vienna, Austria, 2023) with the following packages: lme4 (version 1.1-34), lmerTest (version 3.1-3), emmeans (version 1.8.7), and geepack (version 1.3-9).

#### 2.17.1. Experiment 1 (Semen Storage—Continuous Outcomes: CASA, Antioxidant Levels)

Outcome variables: TM, PM, VSL, VCL, ALH, BCF, ATP, T-AOC, MDA, ROS, *CAT*, *SOD*, GSH-Px.

Model: Linear mixed-effects model (LMM) with restricted maximum likelihood (REML).

Fixed effects: Formulation (control vs. treatment), storage day (0, 5, 10, 15), and formulation x day interaction.

Random effects: Bull (intercept) and ejaculate nested within bull (intercept).

Repeated measure: Storage day within aliquot.

Correlation structure: Unstructured covariance matrix for repeated measures within aliquot.

Planned contrasts: Pairwise comparison of formulations at each time point, adjusted by Tukey’s method.

Model assumptions: Residual normality assessed by Shapiro–Wilk test and Q-Q plots; homoscedasticity assessed by Levene’s test; outliers identified by Cook’s distance.

Missing data: No missing data for Experiment 1.

Significance level: Alpha = 0.05 (two-tailed).

#### 2.17.2. Experiment 1 (Semen Storage—Proportion Outcomes: Acrosome Integrity, Plasma Membrane Integrity, MMP, DNA Integrity)

Model: Generalized linear mixed model (GLMM) with binomial distribution and logit link.

Outcome: Proportion of intact cells per field.

Fixed effects: Formulation, day, formulation x day.

Random effects: Bull, ejaculate within bull, field within slide.

Weights: Number of cells counted per field.

Planned contrasts: Odds ratios for formulation effect at each day.

#### 2.17.3. Experiment 2 (IVF Outcomes)

Outcomes: Cleavage rate per oocyte; blastocyst rate per oocyte and per cleaved embryo.

Model: GLMM with binomial distribution and logit link.

Fixed effect: Formulation.

Random effects: IVF session (intercept), semen batch (intercept).

Weights: Number of oocytes per session.

Planned contrast: Odds ratio for treatment vs. control.

#### 2.17.4. Experiment 3 (AI Pregnancy Outcome)

Primary analysis (intention-to-treat): All allocated cows included.

Fixed effects: Formulation, parity, lactation status, body condition score, days postpartum, inseminator.

Random effects: Farm batch (intercept), bull/semen batch (intercept).

Planned contrast: Odds ratio for treatment vs. control, with 95% confidence interval.

Secondary analysis: Conditional analysis restricted to inseminated cows.

#### 2.17.5. Gene Expression (qPCR)

Model: LMM on deltaCt values.

Random effects: Bull, ejaculate within bull.

Reference gene: GAPDH; stability confirmed by geNorm algorithm (M < 0.5).

#### 2.17.6. Multiplicity and Effect Reporting

No formal multiplicity adjustment was applied across outcomes (see Section 2.1.6).

All models were checked for convergence, singularity, and overdispersion (where applicable).

Results are reported as estimated marginal means (or odds ratios for binomial outcomes) with 95% confidence intervals and exact *p*-values.

## 3. Results

### 3.1. Effects of Treatment Formulation on Kinetic Parameters of Bull Sperm

The results are presented in Table 4. The treatment formulation showed more favorable sperm kinetic parameters than the control formulation during storage at 0 °C (Table 4). Formulation effect (main effect): TM (*p* = 0.018), PM (*p* = 0.024), and VCL (*p* = 0.031) were significantly higher in the treatment formulation across all time points. Time effect: All parameters declined significantly over time (*p* < 0.001 for all). Formulation x time interaction: Significant for TM (*p* = 0.042) and PM (*p* = 0.038), indicating that the difference between formulations was more pronounced at later time points. Time-specific contrasts (Tukey-adjusted):

Day 5: TM (*p* = 0.041), PM (*p* = 0.038), VCL (*p* = 0.029); VSL, ALH, BCF (*p* > 0.05).

Day 10: TM (*p* = 0.012), PM (*p* = 0.015), VSL (*p* = 0.044), VCL (*p* = 0.018); ALH, BCF (*p* > 0.05).

Day 15: TM (*p* = 0.008), PM (*p* = 0.011), VSL (*p* = 0.033), VCL (*p* = 0.014); ALH (*p* = 0.089), BCF (*p* = 0.076).

These results indicate that the treatment formulation improved TM, PM, VSL, and VCL, but did not significantly affect ALH or BCF at any time point after adjustment for multiple comparisons.

### 3.2. Effects of Treatment Formulation on Acrosome and Plasma Membrane Integrity of Bull Sperm

**Figure 1:**
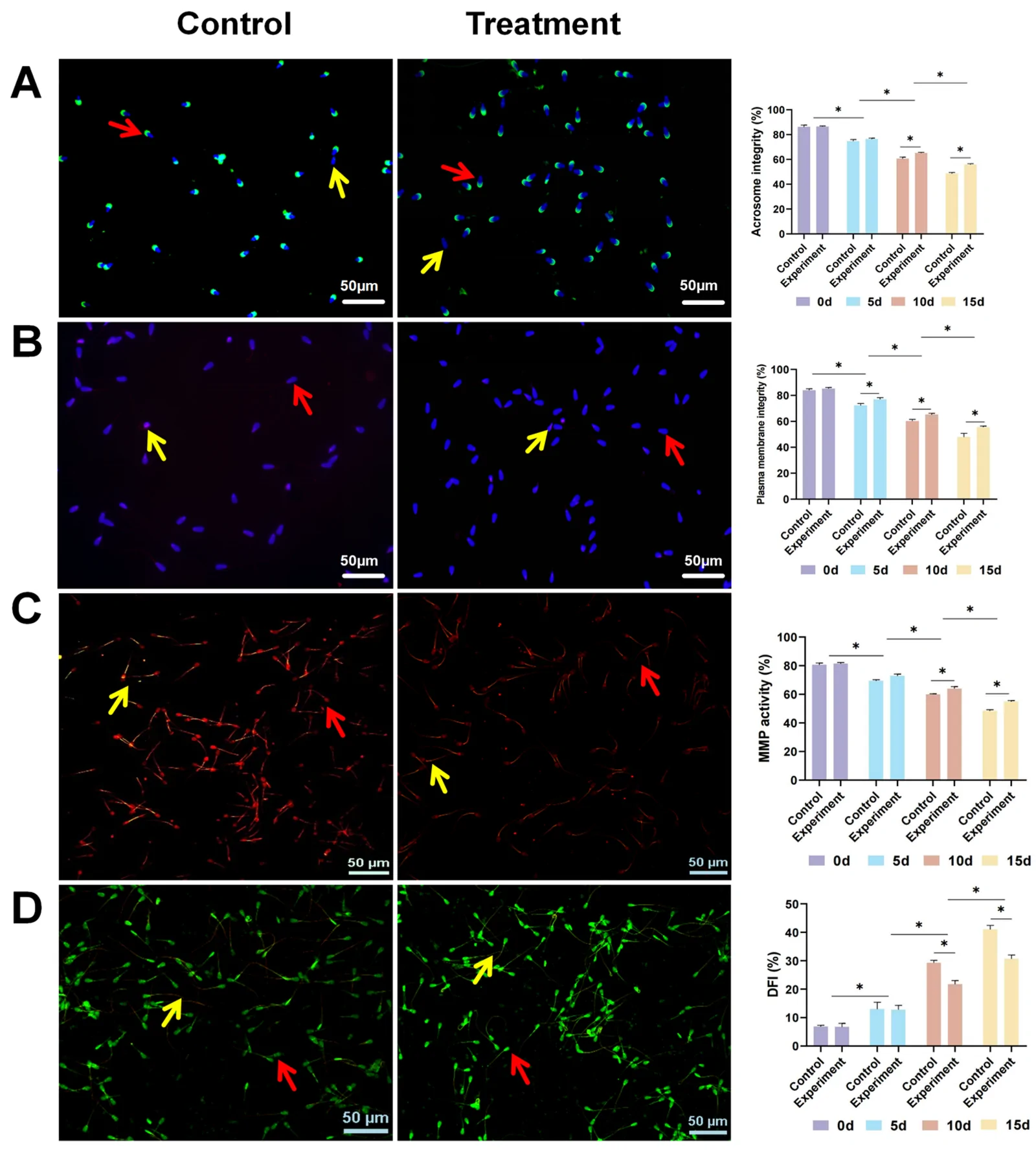
Effects of treatment formulation on the structure of bull sperm preserved at 0 °C. Biological n = 5 bulls per group. For each bull, 2 aliquots were assessed; for each aliquot, ≥3 fields were imaged and ≥200 sperm counted per field. Technical replicates were averaged per bull. Error bars = SD. *p* < 0.05 (GLMM, Tukey-adjusted). (**A**) Effects of different diluents on preserving sperm acrosome integrity. Excited with UV light in the range of 494–518 nm. The emission of FITC-PNA green fluorescence from the apical region of the sperm head indicates intact acrosome. (**B**) Effects of different diluents on the preservation of sperm plasma membrane integrity. Live sperm in suspension were stained with PI (5 µg/mL) and Hoechst 33342 (10 µg/mL). Excitation/emission: Hoechst 405/461 nm (blue, all nuclei); PI 535/617 nm (red, damaged membranes). Sperm heads with intact plasma membranes show blue fluorescence only; red-headed sperm have damaged membranes. Red arrows indicate sperm with intact membranes (blue only); yellow arrows indicate sperm with damaged membranes (red nuclei). (**C**) Effects of different diluents on mitochondrial membrane potential (MMP) of preserved sperm. Live sperm in suspension were stained with JC-1. Excitation/emission: JC-1 aggregates 514/590 nm (red, high MMP); JC-1 monomers 514/529 nm (green, low MMP). MMP declined over time in both groups but remained higher in the treatment formulation at days 10 and 15 (*p* = 0.018 and *p* = 0.012, respectively). (**D**) Effects of different diluents on sperm DNA integrity (AO method). Live sperm in suspension were stained with acridine orange. Excitation/emission: 488/515–530 nm (green, intact DNA); 488 /> 630 nm (red, denatured DNA). Sperm with intact DNA are stained green; red fluorescence represents sperm with denatured DNA. This is not the SCSA DFI method. * indicates a significant difference (*p* < 0.05).

In relation to the control formulation, the treatment formulation exhibited a significant improvement in the integrity of both the acrosome and the sperm plasma membrane (*p* < 0.05) (Figure 1A,B). The findings suggest that the treatment formulation significantly improves the structural integrity of both the acrosome and plasma membrane in sperm stored at 0 °C.

### 3.3. Effects of Treatment Formulation on MMP of Bull Sperm

The mitochondrial membrane potential (MMP) of sperm in the treatment formulation showed a relatively stable pattern and was notably increased compared to the control formulation (*p* < 0.05) (Figure 1C). These results indicate that the treatment formulation effectively maintains sperm MMP.

### 3.4. Effects of Treatment Formulation on DNA Integrity of Bull Sperm

The percentage of AO red-positive sperm (indicative of DNA denaturation) was lower in the treatment formulation than in the control formulation (*p* = 0.032) (Figure 1D). These results indicate that the treatment formulation effectively enhances DNA integrity during sperm preservation, playing a crucial role in maintaining the stability of sperm genetic material. The original images of the experiment are detailed in Supplementary File S1—DNA integrity.

### 3.5. Effects of Treatment Formulation on Ultrastructure of Bull Sperm

**Figure 2:**
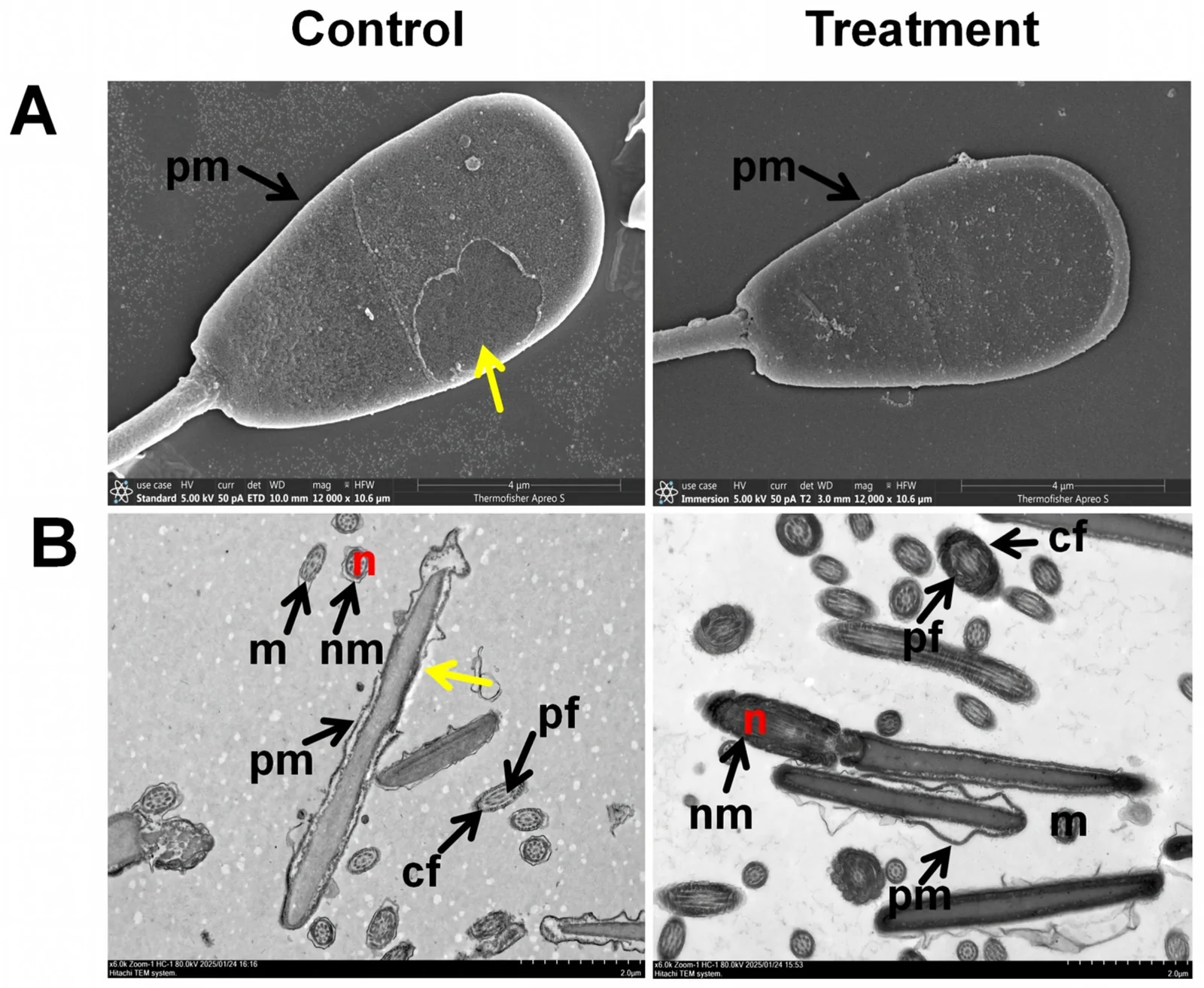
Effects of treatment formulation on the ultrastructure of bull sperm preserved at 0 °C (n = 5). (**A**) Scanning electron microscopy (Thermo Fisher Apreo S, 5 kV) revealed the effects of different diluents on surface structures such as the sperm cell membrane. (**B**) Transmission electron microscopy (Hitachi HT7800, Tokyo, Japan, 80 kV) revealed the effects of different diluents on the internal structure of sperm. In the figures, yellow arrows denote damage to the detected indicators. pm: plasma membrane; pf: peripheral fibers; m: mitochondria; cf: central fibers; n: nucleus; nm: nuclear membrane.

Representative SEM and TEM images qualitatively suggested better preservation of some membrane and ultrastructural features in the treatment formulation (Figure 2). SEM images showed that the plasma membrane of sperm heads in the treatment formulation appeared more intact in the representative fields examined, whereas the control formulation showed varying degrees of membrane damage in some cells. TEM images suggested that mitochondria and axonemal structures in the treatment formulation appeared better preserved in the limited sample examined. These observations are illustrative only and cannot be generalized to the full sample. No quantitative ultrastructural scoring was performed.

### 3.6. Effects of Treatment Formulation on Antioxidant Levels of Bull Sperm

**Figure 3:**
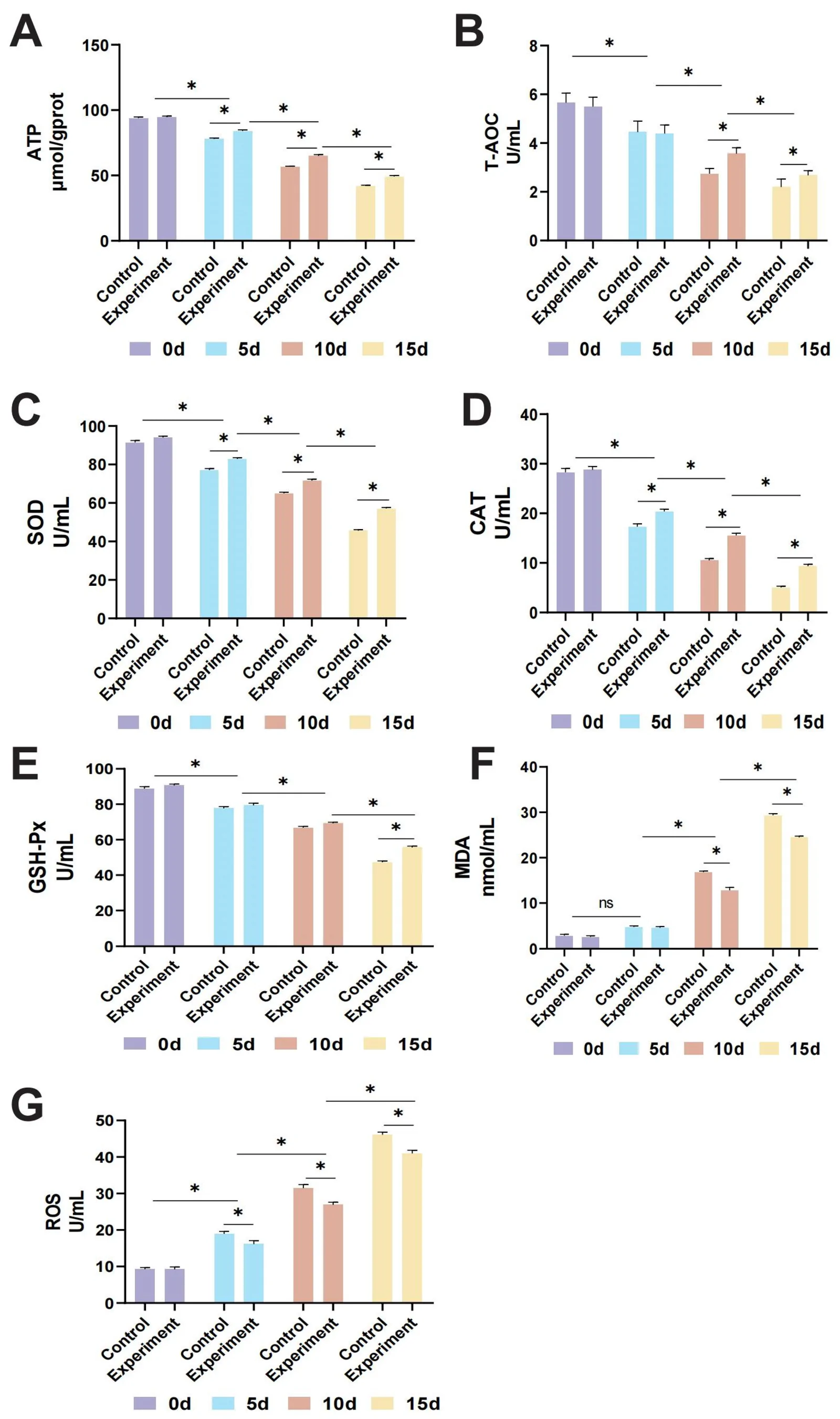
Effects of different diluents on the antioxidant levels of bull sperm preserved at 0 °C. (**A**–**G**) The effects on sperm ATP level, total antioxidant capacity, antioxidant enzyme activities, MDA concentrations, and ROS level were systematically examined at storage intervals of 0, 5, 10, and 15 days. Biological n = 5 bulls per group × 2 aliquots per bull × 4 time points = 40 samples. Technical n = 3 replicate measurements per sample. Technical replicates were averaged before analysis. Error bars = SD. * indicates a significant difference (*p* < 0.05). ns, not significant.

Compared to the control formulation, the treatment formulation showed higher levels of ATP, total antioxidant capacity, and antioxidant enzyme activities in the semen-extender mixture (*p* < 0.05) (Figure 3A–E), while MDA and ROS levels were lower (*p* < 0.05) (Figure 3F,G). Because sperm were not separated from the extender, these differences reflect the antioxidant capacity of the entire mixture, which includes contributions from extender components (vitamin C, GSH, BSA, fructose) as well as sperm-derived factors.

### 3.7. Effects of Treatment Formulation on the Expression of Sperm Antioxidant and Apoptosis-Related Genes

**Figure 4:**
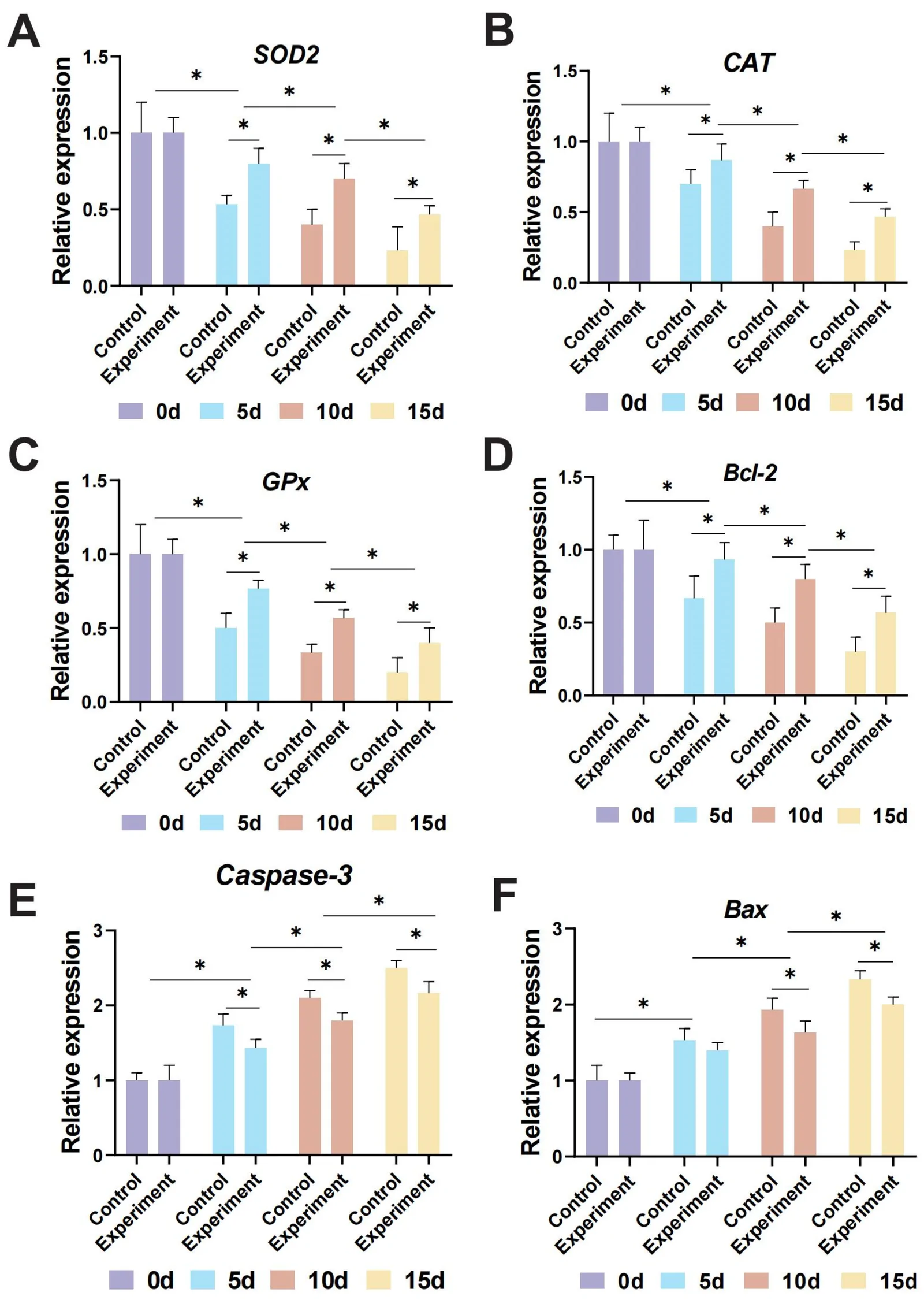
Effects of different diluents on the expression of antioxidant and apoptosis-related genes in bull sperm stored at 0 °C. (**A**–**F**) The expression levels of antioxidant genes *SOD2*, *CAT*, and *GPx*, as well as the anti-apoptotic gene *Bcl-2* and the apoptotic genes *Caspase-3* and *Bax*, were measured at storage intervals of 0, 5, 10, and 15 days. Biological n = 5 bulls per group × 2 aliquots per bull × 4 time points = 40 samples. Technical n = 3 replicate measurements per sample. Technical replicates were averaged before analysis. Error bars = SD. * indicates a significant difference (*p* < 0.05).

The sperm of the treatment formulation exhibited significantly elevated relative abundance of transcripts for the antioxidant genes *SOD2, CAT*, and *GPx*, along with the anti-apoptosis-related gene *Bcl-2*, compared to the control formulation (*p* < 0.05) (Figure 4A–D). However, the relative abundance of pro-apoptosis-related transcripts *Caspase-3* and *Bax* was significantly reduced (Figure 4E,F). These findings reflect differences in the relative abundance of selected apoptosis-related transcripts and do not demonstrate actual apoptotic activity, which would require direct apoptosis assays (e.g., Annexin V, TUNEL).

### 3.8. Effects of Treatment Formulation on IVF Ability of Bull Sperm

The results of IVF in cattle are presented in Table 5, the cleavage rate was 69.03% (156/226) in the control formulation and 74.78% (172/230) in the treatment formulation. The blastocyst rate per cleaved embryo was 26.28% (41/156) vs. 30.23% (52/172). The blastocyst rate per cultured oocyte was 18.14% (41/226) vs. 22.61% (52/230). None of these differences were statistically significant after accounting for session and semen batch clustering (cleavage: OR 1.34, 95% CI 0.87–2.07, *p* = 0.189; blastocyst per oocyte: OR 1.24, 95% CI 0.78–1.97, *p* = 0.371).

### 3.9. Effects of Treatment Formulation on Pregnancy Rate in AI of Bull Sperm

The results of synchronized estrus in cattle are presented in Table 6. The treatment formulation and control formulation had similar estrus detection rates (91.58% vs. 92.38%). In the intention-to-treat analysis, the pregnancy rate was 63.16% (120/190) in the treatment formulation and 59.05% (124/210) in the control formulation (adjusted OR 1.18, 95% CI 0.78–1.78, *p* = 0.428). In the conditional analysis restricted to inseminated cows, the pregnancy rate was 68.97% (120/174) vs. 63.92% (124/194) (adjusted OR 1.25, 95% CI 0.81–1.93, *p* = 0.312).

## 4. Discussion

The efficient preservation technology of bull semen not only enhances reproductive efficiency but also provides critical support for genetic resource conservation, superior genotype selection, and modern livestock industry development [22]. However, during in vitro preservation, semen quality deteriorates due to multifaceted factors, leading to compromised physiological functions and structural integrity [23]. This study investigates the efficacy of the treatment formulation in maintaining bull semen quality during 0 °C preservation. To our knowledge, this is the first study to systematically evaluate its role in the low-temperature preservation of bull semen.

Sperm viability and kinematic parameters serve as critical biomarkers for both fertilization competence and embryonic developmental potential [24]. Soy lecithin has been investigated as an egg-yolk alternative in semen extenders due to its phospholipid content, which may interact with sperm membranes [25]. In the meantime, the observed differences between the two formulations may be related to multiple factors, including the lipid source (soy lecithin vs. egg yolk), the absence of egg yolk, and potential differences in the physical properties of the mixtures. We cannot attribute the findings to any single component. Fructose is a common energy substrate in semen extenders that may support sperm metabolism during liquid storage [26], but both formulations contained identical concentrations of fructose, ATP, GSH, vitamin C, and other non-lipid components; therefore, differences in these components cannot explain the observed outcomes. Tris and BSA are standard components of semen extenders that contribute to osmotic regulation and pH buffering [27]. Our findings indicate significant improvements in TM, PM, VSL, and VCL. ALH and BCF showed numerical differences favoring the treatment formulation but did not reach statistical significance after adjustment. These findings indicate that utilizing a treatment formulation formulated with plant-derived soy lecithin for the preservation of bull sperm is significantly more effective than control formulation.

The acrosome, as a pivotal structure during sperm–egg fusion, requires structural integrity for successful fertilization [28]. Simultaneously, plasma membrane integrity serves as a critical determinant of sperm functionality and fertilization competence [29]. The findings of this research demonstrate that the concentrated-diluent group exhibited significantly greater acrosomal and plasma membrane integrity compared to the control formulation, with particularly notable differences observed in the SEM and TEM analyses. One possible explanation for the observed differences is that the lipid matrix (soy lecithin vs. egg yolk) interacts differently with sperm membranes and antioxidant components. The protective effect of low temperature may be due to the synergistic effect of antioxidants (vitamin C, GSH) and trehalose in the diluent. These components may neutralize ROS generated during storage, thereby mitigating oxidative damage to acrosomal and plasma membranes [30]. This is consistent with the results of this study on the antioxidant enzyme activity in bull semen.

Mitochondria, functioning as cellular power plants, require stable membrane potential to sustain sperm bioenergetics and viability [31]. The experimental results showed that MMP was significantly higher in the concentrated-diluent group than in the control formulation. This protective effect arises from the capability of key components of the diluent to regulate redox homeostasis within mitochondria, thereby mitigating oxidative stress damage in sperm cells [32,33].

DNA damage frequently occurs during the in vitro preservation of semen, potentially resulting in fertilization failure or abnormal embryonic development [34]. The experimental results demonstrated that the level of sperm DNA damage retained in the treatment formulation was significantly lower than that observed in the control formulation. This finding suggests that the treatment formulation plays a role in mitigating the accumulation of DNA damage. Multiple antioxidant components likely reduced oxidative attack on DNA through free radical scavenging [35]. Vitamin C is an antioxidant commonly included in semen extenders and may further enhance cellular redox homeostasis, synergistically protecting genomic integrity [36].

Strategic optimization of diluent formulation and preservation protocols significantly enhanced sperm kinematic parameters and post-thaw viability, thereby improving both pregnancy rates and economic benefits in AI programs [37]. The findings of this research showed a notable improvement in both the cleavage rate and the blastocyst rate within the group that utilized the treatment formulation. Furthermore, the treatment formulation significantly improved the pregnancy rate. We did not include a cryopreserved semen control formulation; therefore, no comparison with cryopreservation can be made. The fertility findings from IVF and AI showed numerical differences favoring the treatment formulation, but these were not statistically significant after appropriate cluster-aware analysis. These preliminary findings require confirmation in a fully reported, randomized controlled trial.

This study has several limitations that affect generalizability. First, the semen storage experiment used only five bulls from a single breed and location; results may not extend to other breeds, ages, or environmental conditions. Second, all laboratory procedures were performed at a single institution, which may introduce laboratory-specific technical variation. Third, the AI field study was conducted at one commercial farm with a specific herd management system. Fourth, the absence of formal sample-size calculations and multiplicity adjustments means that statistical findings should be considered exploratory. Finally, the AI study used a non-randomized allocation rule, which may introduce selection bias despite adjustment for recorded confounders.

## 5. Conclusions

The treatment formulation developed in this study can be used for the preservation of bull semen at 0 °C. The diluent shows significant advantages in maintaining sperm viability, structural integrity, and fertility. This study provides new insights into the efficient preservation of bull semen and the development of breeding techniques.

## Figures and Tables

**Table 1:** Preparation method of the control formulation.

Raw Material	Content
fructose	2.00 g
tris	4.84 g
bovine serum albumin (BSA)	0.18 g
adenosine triphosphate (ATP)	0.08 g
reduced glutathione (GSH)	0.11 g
sodium citrate	2.66 g
vitamin C	0.55 g
trehalose	0.50 g
penicillin	0.74 g
egg yolk	40 mL
ddH_2_O	136 mL

**Table 2:** (**A**) Preparation method of the treatment formulation. (**B**) Stepwise preparation of control and treatment formulations.

(**A**)			
**Raw Material**			**Content**
soy lecithin			0.75 g
fructose			2.00 g
tris			4.84 g
bovine serum albumin (BSA)			0.18 g
adenosine triphosphate (ATP)			0.08 g
reduced glutathione (GSH)			0.11 g
sodium citrate			2.66 g
vitamin C			0.55 g
trehalose			0.50 g
penicillin			0.74 g
ddH_2_O			35.2 mL
(**B**)			
**Step**	**Parameter**	**Control Formulation**	**Treatment Formulation**
Stock solution	Composition	All components (Table 1) dissolved in 136 mL ddH_2_O + 40 mL egg yolk	All components (Table 2) dissolved in 35.2 mL ddH_2_O + 0.75 g soy lecithin
	Total stock volume	176 mL	35.2 mL
	Storage	4 °C, prepared fresh before each experiment	4 °C, prepared fresh before each experiment
Reconstitution	Procedure	No dilution required; used directly as working solution	No pre-dilution before contact with sperm. The concentrated formulation was used directly. The term ‘concentrated’ refers to the higher component concentrations per unit volume achieved by reducing the water and removing the egg yolk, not to a five-fold stock that requires dilution.
	Volume of water added before use	0 mL	0 mL
Final working solution	Volume	176 mL per batch	35.2 mL per batch
	pH	6.8 ± 0.1	6.8 ± 0.1
	Osmolality	310 ± 10 mOsm/kg	310 ± 10 mOsm/kg
	Egg yolk concentration	22.7% (*v*/*v*)	0%
	Soy lecithin concentration	0 g/100 mL	2.13 g/100 mL
Final semen mixture	Semen:extender ratio	1:4 (*v*/*v*)	1:4 (*v*/*v*)
	Total final volume per aliquot	20 mL	20 mL
	Final sperm concentration	1 × 10^9^/mL	1 × 10^9^/mL
	Final AI dose volume	0.25 mL	0.25 mL
	Final AI dose sperm number	2 × 10^7^ sperm	2 × 10^7^ sperm

The treatment formulation was not diluted five-fold before contact with sperm. The five-fold concentration factor refers to the reduction in total liquid volume (from 176 mL to 35.2 mL) achieved by removing egg yolk and reducing water, while maintaining the same absolute mass of non-lipid components per final semen mixture. Both formulations were mixed with semen at an identical 1:4 ratio to produce the same final component concentrations in the semen-extender mixture, with the exception that the lipid source differed (egg yolk vs. soy lecithin).

**Table 3:** Primer sequence information.

Genes	Primer Sequences (5′ → 3′)	Annealing Temperature (°C)	Product Length (bp)
*GAPDH*	F:CCCCAACGTGTCGGTTGT R:CTCGGACGCCTGCTTCAC	59	91
*SOD2*	F:TTTGTAGGAGCGCCGAATAC R:TAACCTCCTGGCTCTTTCCA	59	217
*CAT*	F:TGCCCATACTTCCCGTCC R:GGTCCAGGTTACCGTCAG	59	172
*GPx*	F:CAGGTACAGCCGTCGCTTTC R:AAAATCCCGAGAGTAGCATC	59	137
*Bcl-2*	F: AGGGCATTCAGTGACCTGAC R: CGATCCGACTCACCAATACC	59	193
*Bax*	F: TGCCTCAGGATGCATCTACC R: AAGTAGAAAAGCGCGACCAC	55	199
*Caspase-3*	F:CAGACAGTGGTGCTGAGGATGA R:GCTACCTTTCGGTTAACCCGA	59	73

**Table 4:** Effects of the treatment formulation on sperm kinetic parameters preserved at 0 °C.

CASA Parameters	TM (%)		PM (%)		VSL (μm/s)		VCL (μm/s)		ALH (μm)		BCF (Hz)	
Groups	Control	Treatment	Control	Treatment	Control	Treatment	Control	Treatment	Control	Treatment	Control	Treatment
0 d	92.46 ± 2.65 ^A^	92.68 ± 2.38 ^A^	82.37 ± 2.02 ^A^	83.11 ± 2.14 ^A^	72.19 ± 2.21 ^A^	73.33 ± 1.87 ^A^	112.45 ± 5.16 ^B^	114.62 ± 6.47 ^B^	8.19 ± 2.21 ^A^	8.33 ± 1.87 ^A^	28.32 ± 3.41 ^A^	29.07 ± 2.94 ^A^
5 d	80.77 ± 2.21 ^bB^	85.56 ± 2.44 ^aB^	69.58 ± 2.35 ^bB^	75.09 ± 1.94 ^aB^	58.42 ± 2.41 ^B^	61.48 ± 2.03 ^B^	124.42 ± 3.83 ^bA^	132.65 ± 5.14 ^aA^	6.63 ± 1.66 ^B^	6.71 ± 2.02 ^B^	18.48 ± 2.17 ^B^	20.36 ± 2.24 ^B^
10 d	64.26 ± 4.11 ^bC^	72.18 ± 3.04 ^aC^	53.62 ± 3.06 ^bC^	62.89 ± 2.53 ^aC^	47.62 ± 2.17 ^bC^	54.22 ± 2.93 ^aC^	97.57 ± 5.24 ^bC^	109.26 ± 4.13 ^aC^	4.77 ± 2.13 ^C^	5.12 ± 2.84 ^C^	14.82 ± 2.17 ^B^	15.49 ± 2.88 ^B^
15 d	49.52 ± 3.88 ^bD^	56.76 ± 2.64 ^aD^	41.72 ± 5.13 ^bD^	50.76 ± 2.45 ^aD^	36.77 ± 2.25 ^bD^	42.65 ± 2.68 ^aD^	73.59 ± 4.45 ^bD^	86.19 ± 6.28 ^aD^	3.29 ± 1.46 ^D^	3.84 ± 1.44 ^D^	8.59 ± 1.77 ^C^	11.06 ± 1.82 ^C^

Note: Biological n = 5 bulls; ejaculates = 5 (1 per bull); aliquots = 10 (2 per ejaculate); fields ≥3 per slide; sperm counted = ≥200 per field. Technical replicates (fields and cells) were averaged per aliquot before statistical analysis. Values are mean ± SD. Statistical model: linear mixed-effects model with bull and ejaculate as random effects. Time-specific pairwise comparisons (Tukey-adjusted): *p* < 0.05 for treatment vs. control. ALH and BCF showed no significant between-group differences at any time point. Different superscripts A–D within a row indicate significant differences between values (*p* < 0.05). Different superscripts a, b within a column indicate significant differences between values (*p* < 0.05).

**Table 5:** Effects of treatment formulation on the IVF ability of preserved bull sperm.

Groups	Replicates	Oocyte Number	Cleaved Embryos (n)	Cleavage Rate (%)	Blastocysts per Cleaved Embryo (n)	Blastocyst Rate per Cleaved (%)	Blastocysts per Oocyte (n)	Blastocyst Rate per Oocyte (%)
Control	5	226	156	69.03	41	26.28	41	18.14
Treatment	5	230	172	74.78	52	30.23	52	22.61

Note: The IVF treatment comprised five independent sessions (n = 5). Values are reported as counts with percentages. Odds ratio (treatment vs. control) for cleavage: 1.34 (95% CI: 0.87–2.07), *p* = 0.189. Odds ratio for blastocyst per oocyte: 1.24 (95% CI: 0.78–1.97), *p* = 0.371. Odds ratio for blastocyst per cleaved embryo: 1.19 (95% CI: 0.72–1.96), *p* = 0.498. Biological n = 5 IVF sessions. Semen batches = 5 per formulation (linked to bull). Oocytes: 226 control, 230 treatment. Technical replicates = wells/drops per session.

**Table 6:** Effects of treatment formulation on pregnancy rate of preserved bull sperm.

Groups	Replicates	Cows Enrolled	Cows Synchronized	Cows Allocated	Cows Showing Estrus	Cows Inseminated	Pregnant (ITT)	Pregnant (Conditional)
Control	5	210	210	210	194 (92.38%)	194	124/210 (59.05%)	124/194 (63.92%)
Treatment	5	190	190	190	174 (91.58%)	174	120/190 (63.16%)	120/174 (68.97%)

Note: The AI experiment comprised five independent batches (n = 5). The individual cow was the experimental unit. Intention-to-treat pregnancy rate: control 124/210 (59.05%), treatment 120/190 (63.16%). Odds ratio (treatment vs. control, adjusted for batch): 1.18 (95% CI: 0.78–1.78), *p* = 0.428. Conditional pregnancy rate (among inseminated cows): control 124/194 (63.92%), treatment 120/174 (68.97%). Odds ratio: 1.25 (95% CI: 0.81–1.93), *p* = 0.312. Biological n = 400 cows. Batches = 5. Bulls = 5 (pooled). Inseminators = 3.

## Data Availability

All the data supporting the results of this study can be found in the article. The data that is not publicly available is the core data of the ranch and is not provided for public use.

## Supplementary Materials

The following supporting information can be downloaded at: https://www.mdpi.com/article/10.3390/biology15181577/s1.
